# Receptor-Like Kinases BAK1 and SOBIR1 Are Required for Necrotizing Activity of a Novel Group of *Sclerotinia sclerotiorum* Necrosis-Inducing Effectors

**DOI:** 10.3389/fpls.2020.01021

**Published:** 2020-07-10

**Authors:** Shirin Seifbarghi, Mohammad Hossein Borhan, Yangdou Wei, Lisong Ma, Cathy Coutu, Diana Bekkaoui, Dwayne D. Hegedus

**Affiliations:** ^1^ Agriculture and Agri-Food Canada, Saskatoon, SK, Canada; ^2^ Department of Biology, University of Saskatchewan, Saskatoon, SK, Canada; ^3^ Department of Food and Bioproduct Sciences, University of Saskatchewan, Saskatoon, SK, Canada

**Keywords:** *Sclerotinia sclerotiorum*, cell death, necrosis, receptors, virus-induced gene silencing

## Abstract

*Sclerotinia sclerotiorum* is a characteristic necrotrophic plant pathogen and is dependent on the induction of host cell death for nutrient acquisition. To identify necrosis-inducing effectors, the genome of *S. sclerotiorum* was scanned for genes encoding small, secreted, cysteine-rich proteins. These potential effectors were tested for their ability to induce necrosis in *Nicotiana benthamiana via Agrobacterium*-mediated expression and for cellular localization in host cells. Six novel proteins were discovered, of which all but one required a signal peptide for export to the apoplast for necrotizing activity. Virus-induced gene silencing revealed that the five necrosis-inducing effectors with a requirement for secretion also required the plant co-receptor-like kinases Brassinosteroid Insensitive 1-Associated Receptor Kinase 1 (BAK1) and Suppressor of BAK1-Interacting Receptor-like Kinase 1 (SOBIR1) for the induction of necrosis. *S. sclerotiorum* necrosis-inducing effector 2 (SsNE2) represented a new class of necrosis-inducing proteins as orthologs were identified in several other phytopathogenic fungi that were also capable of inducing necrosis. Substitution of conserved cysteine residues with alanine reduced, but did not abolish, the necrotizing activity of SsNE2 and full-length protein was required for function as peptides spanning the entire protein were unable to induce necrosis. These results illustrate the importance of necrosis-inducing effectors for *S. sclerotiorum* virulence and the role of host extracellular receptor(s) in effector-triggered susceptibility to this pathogen.

## Introduction

The infection of a host plant by a fungal pathogen most often begins with an attempt to penetrate the cuticle. Beyond this barrier, pathogens face a multi-faceted defense system that restricts further growth and dissemination. To overcome plant defenses, pathogens secrete an array of effector proteins to suppress the host immune system and to reprogram the expression of host genes involved in signaling, cell structure and metabolism (Dalio et al., 2018). Characterizing these effectors, determining their targets and understanding their interaction with host defenses is key to uncovering pathogenicity/virulence mechanisms and to devising strategies to control diseases.

In addition to mechanical and cellular defense barriers conferring passive resistance, pathogens encounter a series of active defense mechanisms triggered by the perception of conserved pathogen-associated molecular patterns (PAMPs), such as lipopolysaccharides, flagellin and chitin, termed PAMP-triggered immunity (PTI). Multiple plasma membrane-localized pattern recognition receptors (PRR), such as membrane-associated receptor-like kinases (RLK) or receptor-like proteins (RLP), can be engaged in pathogen recognition (Saijo et al., 2018). RLKs have an extracellular domain, a transmembrane (TM) domain and an intracellular kinase domain, while RLPs lack the kinase domain. These, together with Brassinosteroid Insensitive 1-Associated Receptor Kinase 1 (BAK1)/Somatic Embryogenesis Receptor Kinase 3 (SERK3), mediate PAMP recognition (Cook et al., 2015; Khaled et al., 2015). Pathogens also release an array of effector proteins that may prevent PAMP recognition or interfere with consequent signaling (Jones and Dangl, 2006; de Jonge et al., 2010; Dou and Zhou, 2012; Liebrand et al., 2014). In turn, effectors can be recognized by receptors known as resistance (R) proteins (Jones and Dangl, 2006). Effector recognition often results in a hypersensitive reaction (HR) that can restrict biotrophic and hemibiotrophic pathogens, this is known as effector-triggered immunity (ETI) (Jones and Dangl, 2006). Conversely, for necrotrophic pathogens the induction of cell death responses, such as the HR, by effectors provides resources to fuel colonization of the host, a phenomenon that may be referred to as a form of effector-triggered susceptibility (ETS) (Figueroa et al., 2015; Liang and Rollins, 2018; Wang and Wang, 2018). Recognition of PAMPs by PRRs and effectors by R proteins share some common components (Ma and Borhan, 2015). For example, BAK1 and Suppressor of BAK1-Interacting Receptor-like Kinase 1 (SOBIR1) are required for HR induction and ETI triggered by the recognition of effectors (avirulence proteins) by the R proteins *Cladosporium fulvum* 4 (Cf4) from tomato and *Leptosphaeria maculans* Resistance 3 (LepR3) from *B. napus* (Liebrand et al., 2013; Ma and Borhan, 2015; Postma et al., 2015). Similarly, RLP30, SOBIR1 and BAK1, are required for recognition of an elicitor from *Sclerotinia sclerotiorum*, called Sclerotinia culture filtrate elicitor 1 (SCFE1) (Zhang et al., 2013).

Sequencing the genomes of plant pathogens has enabled *in silico* approaches for predicting their effector repertoires (Sperschneider et al., 2015a; Dalio et al., 2018). The general primary criteria for *in silico* identification of effectors includes being relatively small, the presence of a signal peptide (SP), elevated cysteine content, and the absence of TM domains or membrane anchors (Kamoun, 2006; Liu et al., 2012; Lyu et al., 2015). The SP is essential as it mediates secretion of effectors into the host (von Heijne, 1998) and disulfide bonds between cysteine residues are suggested to enhance stability in the host apoplast (Tuori et al., 2000). Effectors target diverse host cellular functions (Toruno et al., 2016) and are generally classified as apoplastic (function in the host extracellular space) or cytoplasmic (function inside host cells) (Stotz et al., 2014). Previous studies showed that fungal and oomycete effector targets are located in a wide variety of subcellular compartments, including the PM, tonoplast, vacuole, endoplasmic reticulum (ER), nucleus and cytosol (Bozkurt et al., 2012; Caillaud et al., 2012; McLellan et al., 2013). Therefore, the expression profile during host invasion, prediction of subcellular localization, and structural properties are important features for the identification of candidate effectors (Dalio et al., 2018). Ultimately, functional validation with stable transgenic plants or transient expression using *Agrobacterium tumefaciens*-mediated expression (agro-expression) and silencing of effectors and their host targets, provides further evidence for effector function and contribution to infection (Sparkes et al., 2006; Mesarich et al., 2014).

*S. sclerotiorum* causes stem rot, one of the most devastating diseases of canola. It is a necrotrophic pathogen that switches to a highly aggressive invasive phase soon after host cuticle penetration aided by the secretion of acids and hydrolytic enzymes (Hegedus and Rimmer, 2005). Recent studies, however, have provided evidence for a two-phase infection model for this pathogen with a brief biotrophic phase occurring soon after penetration (Kabbage et al., 2015; Liang and Rollins, 2018). According to these studies, *S. sclerotiorum* first suppresses plant defense systems during the biotrophic phase followed by rapid induction of host cell death at the onset of the necrotrophic phase (Liang and Rollins, 2018). Seifbarghi et al. (2017) suggested that the biotrophic stage might occur within the first 24 h post-infection since biotrophy-related candidate effector genes, such as those encoding the Lysin Motif (LysM) domain protein and salicylate hydroxylase, were up-regulated during this period. To date, much effort has been directed toward understanding the interaction of *S. sclerotiorum* with its various hosts, which has cast light on the complexity of *S. sclerotiorum*-host interactions. For example, 78 candidate effectors were identified in the genome of *S. sclerotiorum* using various computational tools (Guyon et al., 2014). Some of these play crucial roles during different stages of *S. sclerotiorum* infection; however, the exact functions of most of these effectors remain unknown. Several *S. sclerotiorum* effectors involved in pathogenesis or virulence, including two necrosis and ethylene-inducing proteins (SsNep1 and SsNep2) (Bashi et al., 2010), a protein with a CyanoVirin-N Homology domain (SsCVNH) (Lyu et al., 2015), a secreted integrin-like protein (SSITL) (Zhu et al., 2013), chorismate mutase (SsCM1) (Kabbage et al., 2013), small secreted virulence-related protein 1 (SsSSVP1) (Lyu et al., 2016), BAX inhibitor-1 (SsBI1) (Yu et al., 2015), compound appressorium formation-related protein 1 (SsCaf1) (Xiao et al., 2014), catalase (SsCat1) (Yarden et al., 2014), superoxide dismutase (SsSodI) (Xu and Chen, 2013), cerato-plantanin effector (SsCP1) (Yang et al., 2018a), and a protein similar to a protein elicitor from *Magnaporthe grisea* (SsPemG1) (Pan et al., 2015) have been characterized.

Hemibiotrophs and necrotrophs use effectors to regulate host cell death to their benefit and these can serve as virulence factors in these pathogens (Dickman et al., 2001; Qutob et al., 2002). In this study, bioinformatics and transcriptomic approaches were used to identify *S. sclerotiorum* effector proteins. The necrotizing activity of the candidate effectors was examined by *in planta* transient expression and subcellular localization studies conducted to facilitate identification of host targets. This study revealed that the receptor-like kinases BAK1 and SOBIR1 were required for necrotizing activity of several novel *S. sclerotiorum* necrosis-inducing effectors. This study highlights the contribution of necrosis-inducing effectors to *S. sclerotiorum* virulence and their possible use in targeted effector-guided breeding to identify germplasm with improved resistance to this pathogen.

## Materials and Methods

### Bioinformatics Analysis

*S. sclerotiorum* isolate 1980 was used in this study as the genome sequence of this strain is available. The *S. sclerotiorum* genome sequence annotated by Amselem et al. (2011) was used, although this was revised by Derbyshire et al. (2017) after initiation of this work. The genome-wide search for candidate effectors was conducted using a general pipeline for *in silico* prediction of pathogen effectors (Sperschneider et al., 2015b). The bioinformatic tools used to identify candidate effectors were SignalP 4.1 (Petersen et al., 2011) for predicting the presence of a SP, TMHMM v2.0 (Chen et al., 2003) for TM helixes, Big-PI predictor (Eisenhaber et al., 1998) for glycosylphosphatidylinositol (GPI)-anchored proteins and TargetP 1.1 (Emanuelsson et al., 2007) for subcellular location of proteins. The number of cysteine residues in a given protein was computed using an in-house Perl script and those with more than 2% cysteine residues were retained. Predicted effector proteins smaller than 55 amino acids were excluded from the analysis as the GPI prediction algorithm does not work with smaller proteins. The remaining candidate proteins were then scanned for the presence of an N-terminal [N/L]-[P/I/L]-[I/P]-[R/N/S] (the core canonical NPIR) vacuole localization motif using the CLC Genomics Workbench 7.0.3 (http://www.clcbio.com) motif search tool and proteins without this motif were retained.

The final list of candidate effectors produced from the combination of bioinformatic and transcriptomic methods was subjected to functional annotation using the BLAST2GO plugin (v1.4.4) in CLC Genomics Workbench 8.0.1 (http://www.clcbio.com). The candidate protein sequences were used for searches with BLASTP (https://blast.ncbi.nlm.nih.gov/Blast.cgi) to identify conserved protein domains and homologous proteins in other fungi. WoLF PSORT (Horton et al., 2007) was used to predict the subcellular localization of these proteins. The amino acid sequences were aligned using Clustal omega (https://www.ebi.ac.uk/Tools/msa/clustalo/). Protein structure prediction was conducted using SWISS-MODEL workspace (http://swissmodel.expasy.org/interactive), I-TASSER (https://zhanglab.ccmb.med.umich.edu/I-TASSER/) and Predict Protein (https://www.predictprotein.org/).

### RNA-Seq Analysis

The data from the RNA-Seq study conducted by Seifbarghi et al. (2017) was used to evaluate the *in planta* expression patterns of genes encoding candidate effectors identified by *in silico* analysis during the course of infection. Only candidate effector genes expressed during infection were considered for further analysis.

### Plasmid Construction for Agro- Expression Assays

To clone genes encoding the candidate effectors, cDNA was synthesized using the iScript reverse transcription (RT) supermix available in the RT-qPCR kit (Bio-Rad, CA, USA) following the manufacturer’s instructions. The cDNA was used as a template for PCR amplification of candidate genes to generate amplicons encoding proteins with the SP using corresponding primer pairs F1 and R1, or without the SP using primer pairs F2 and R1 (**Table S1**) to test necrotizing activity. Two additional constructs were made to test the subcellular localization of each protein, one with the SP using primer pairs F1 and R2, and the other without the SP using primer pairs F2 and R2 (**Table S1**). The R2 primers do not encode the stop codon and allow in-frame fusion to Green Fluorescent Protein (GFP). The DNA amplicons were then cloned into the Gateway^®^ entry vector pDONRzeo^©^ (Invitrogen, Carlsbad, USA) following the manufacturer’s protocol. To convert the entry vector into a Gateway^®^ expression vector, candidate genes were transferred from pDONRzeo to pEarleyGate100 (pEG100) and pEarleyGate103 (pEG103) (Earley et al., 2006) using the Gateway protocol (Invitrogen, Carlsbad, USA), and then used for phenotypic and microscopic experiments, respectively. pEG103 provides a C-terminal fusion to GFP.

The cysteine residues in *S. sclerotiorum* Necrosis Effector 2 (SsNE2) were converted to alanine to test the impact of cysteine residues on necrotizing activity. DNA fragments encoding SsNE2 variants with C^38^, C^45^, C^64^, and C^86^ replaced with alanine individually or simultaneously were synthesized and cloned into pEG100 by Thermo Fisher Scientific (Waltham, Massachusetts, USA).

To transiently express orthologous *SsNE2* genes from *Botrytis cinerea* (BCIN_14g01200, GenBank accession XP_001552872.1), *Colletotrichum higginsianum* (CHEC91, GenBank accession XP_018152473.1), *Monilinia fructigena* (DID88_010138, GenBank accession RAL61042.1), and *Fusarium oxysporum* f.sp. *lycopersici* (FOXG_04016, GenBank accession XP_018238493.1), the open reading frames were synthesized and cloned into pEG100 by Thermo Fisher Scientific (Waltham, Massachusetts, USA).

Genes encoding proteins containing different targeting signals, including a nuclear localization signal (NLS), a nuclear export signal (NES), the calcineurin B-like protein 1 myristoylation signal (CBL1) and the Pathogenesis-Related Protein 1 (PR1) signal peptide, were synthesized and cloned into pEG103 (with a C-terminal fusion to GFP) by Thermo Fisher Scientific (Waltham, Massachusetts, USA). Genes encoding proteins fused to GFP along with the C-terminal ER retention signal (KDEL) were synthesized and cloned into pEG100 by Thermo Fisher Scientific (Waltham, Massachusetts, USA). Synthetic DNA sequences are provided in **Table S2**.

### Agro-Expression Assay in *Nicotiana benthamiana*

All constructs were transformed into *A. tumefaciens* strain GV3101. The transformed *A. tumefaciens* colonies were selected on Luria-Bertani (LB) agar containing 10 mg/ml rifampicin and 100 mg/ml kanamycin. Leaves from four to five-week-old *N. benthamiana* plants were infiltrated using an agro-expression technique for both phenotypic and microscopic experiments following the method described by Ma et al. (2012). *A. tumefaciens* was grown in LB-mannitol medium supplemented with 20 μM acetosyringone and 10 mM MES (pH 5.6). Cells were harvested by centrifugation at 2800 *g* for 15 min and then re-suspended in infiltration buffer containing 10 mM MES, 2% w/v sucrose, 0.05% w/v MS salts and 200 μM acetosyringone. After 1 to 2 h, the leaves were pressure-infiltrated with a 1 ml needleless syringe. An *A. tumefaciens* strain harboring the empty vector (AtEV) was used as a negative control, while a strain carrying a construct designed to express the *Phytophthora infestans* Nep1-like protein (PiNPP1.1) was used as a positive control (Kanneganti et al., 2006). The appearance of cell death in infiltrated areas was visually evaluated 3–7 days post-infiltration. Each experiment was conducted at least three times. To assess localization in the ER, *A. tumefaciens* strains harboring pEG103 constructs were co-infiltrated with a second *A. tumefaciens* strain expressing mCherry fused to an ER retention signal (KDEL) (http://www.bio.utk.edu/cellbiol/markers/) at a 1:1 ratio.

### Subcellular Localization of Candidate Effectors *In Planta* Using Confocal Laser-Scanning Microscopy (CLSM)

The *N. benthamiana* leaf cells expressing the GFP fusion proteins were examined using a Zeiss LSM 710 CLSM at 36–48 h post-infiltration. Images were obtained using a 63 X objective lens. An argon laser was used to excite GFP at 488 nm and emission was collected at 493–540 nm. mCherry was excited at 561 nm and emission was collected at 589–754 nm. To detect labeled proteins in the apoplast, small infiltrated leaf sections were treated with KCL (0.85M) for 5 min to induce plasmolysis and then examined using CLSM.

### Plasmid Construction, Expression and Infiltration of Recombinant SsNE2 Protein

To express SsNE2 in *Escherichia coli*, the open reading frame without the region encoding the SP was amplified from cDNA using forward (5’ TGCTCTAGAAATAATTTTGTTTAACTTTAAGAAGGAGATATACCATGGCCACCATTGGACAACGTG 3’) and reverse (5’ CCGCTCGAGCCCAGTCGTAGGTCAAGTCGAAAGCAGT 3’) primers. The resultant PCR product was purified, digested with *XbaI* and *XhoI* restriction enzymes and ligated to pET 28a^+^ linearized with the same enzymes. The construct was verified by sequencing and transformed into *E. coli* SHuffle^®^ T7 (New England Biolabs, Massachusetts, USA). Transformed cells were grown in 10 ml of LB medium containing 50 mg/ml kanamycin at 30°C on a rotary shaker at 220 rpm. After reaching an OD600 of 0.4-0.8, isopropyl β-D-1-thiogalactopyranoside (IPTG) was added to a final concentration of 1 mM to induce expression of the inserted gene. After 3–4 h of incubation at 30°C, cells were harvested by centrifugation at 4,800 *g* at 4ºC for 30 min and then re-suspended in 0.1 M phosphate buffer (pH 7.2) containing 1 mg/ml lysosome and cOmplete™, an EDTA-free protease inhibitor cocktail (Sigma-Aldrich, Ontario, Canada). The suspension was sonicated on ice (3 × 10 s pulses at high intensity with a 10 s cooling period between pulses) to lyse the cells. Cellular debris was removed by centrifugation at 20,000 *g* at 4ºC for 30 min and the supernatant used for further experiments. The presence of recombinant protein was analyzed by SDS-polyacrylamide gel electrophoresis (PAGE) using 12% mini-Protein^®^ precast, stain-free gels (Bio-Rad, CA, USA), and then detected by western blotting using anti-His (C-term)-horse radish peroxidase (HRP) antibody (Invitrogen, Carlsbad, USA).

To test the necrotizing activity of recombinant protein, *E. coli* culture supernatants (ECS) containing SsNE2 were infiltrated into leaves of *N. benthamiana* using a needleless syringe. *E. coli* culture supernatant from a strain carrying the empty vector control strain (ECSEV) was used as a negative control. The development of necrosis symptoms was evaluated daily for seven days after infiltration.

### Synthesis of SsNE2 Truncated Peptides and Infiltration Assay

To assess the involvement of specific peptides derived from SsNE2 in necrotizing activity, truncated peptides were synthesized by Bio Basic (Toronto, Canada) and dissolved in sterile distilled water. Different concentrations of each peptide were tested by infiltrating 25 µg/ml to 1 mg/ml into the leaves of *N. benthamiana* plants using a needleless syringe. Five different peptides were designed and named M1, M2, M3, M1+M2, and M2+M3 (**Table S3**). The development of necrosis symptoms was evaluated daily for seven days after infiltration.

### Virus-Induced Gene Silencing (VIGS) of *BAK1* and *SOBIR1* in *N. benthamiana*

To examine the involvement of BAK1 and SOBIR1 in the necrotizing activity of the necrosis-inducing effectors, VIGS was conducted using a tobacco rattle virus (TRV) based vector system. *A. tumefaciens* GV3101 strains harboring pTRV1 (Ratcliff et al., 2001) and pTRV2 constructs, including *pTRV2:GFP* (Burch-Smith et al., 2006), *pTRV2:PDS* (PDS: phytoene desaturase) (Liu et al., 2002), *pTRV2:NbSOBIR1* (Liebrand et al., 2013) and *pTRV2:NbBAK1* (Chaparro-Garcia et al., 2011), were grown to an OD600 = 1. Each *A. tumefaciens* strain carrying a pTRV2 construct was mixed at a 1:1 ratio with the *A. tumefaciens* strain carrying pTRV1 and infiltrated into leaves of 2-week-old *N. benthamiana* plants similar to agro-expression (Ma et al., 2012).

Three weeks after agro-expression, *N. benthamiana* leaves from three biological replicates infiltrated with the *pTRV2:NbSOBIR1*, *pTRV2:NbBAK1*, or *pTRV2:GFP* constructs, as well as untreated *N. benthamiana* leaves as an un-infected negative control, were collected. Total RNA was extracted and cDNA synthesized as described in Seifbarghi et al. (2017). Quantitative PCR (qPCR) was performed to assess the expression of *BAK1* and *SOBIR1* using a CFX96 real-time PCR machine (Bio-Rad, CA, USA) with SsoAdvanced™ Universal SYBR^®^ Green Supermix (Bio-Rad, CA, USA). The amplification conditions were 30 s at 95° C, followed by 40 cycles of 10 s at 95° C, 30 s at 60° C, and a melt curve of 5 s at 65° to 95° C. The primer pairs used for qPCR were previously described by Liebrand et al. (2013) and Chaparro-Garcia et al. (2011). qPCR data were normalized using expression of the endogenous *actin* gene as a reference. The expression (fold change) was reported using the 2^−ΔΔCt^ method (Livak and Schmittgen, 2001) and compared to the control (*pTRV2:GFP*).

Three weeks after agro-expression with the the *pTRV2:NbSOBIR1, pTRV2:NbBAK1*, or *pTRV2:GFP* constructs, leaves of *N. benthamiana* were infiltrated with *A. tumefaciens* carrying the effector constructs, a Bcl2-Associated protein X construct (BAX; Lacomme and Santa Cruz, 1999) as a BAK1/SOBIR1-independent necrosis-inducting protein positive control or AtEV as a negative control. To examine the involvement of BAK1 and SOBIR1 in the necrotizing activity of recombinant SsNE2, the leaves were infiltrated with ECS containing SsNE2 protein or ECSEV as a negative control. The development of necrosis symptoms was evaluated visually 3-7 days post-infiltration. The experiments were conducted three times.

## Results

### A Catalogue of Putative *S. sclerotiorum* Effectors

As a first step towards the identification of necrosis-inducing effectors, bioinformatic approaches were applied to predict effector proteins encoded by the *S. sclerotiorum* genome. First, a search for the presence of a SP using SignalP 4.1 resulted in 912 genes encoding secreted proteins. Subsequently, proteins with an N-terminal SP were screened for their subcellular localization signals to exclude membrane-bound and membrane-anchored proteins using TargetP, TMHMM, and Big-PI software, respectively. Proteins harbouring an NPIR motif immediately adjacent to the predicted SP cleavage site that are targeted to the vacuole (Carter et al., 2004) were also excluded from the list of candidate effectors. To enrich for potential effectors, only small proteins (55-250 amino acids) with more than 2% cysteine content (105 proteins) were considered as candidate effectors.

### Expression Patterns of Genes Encoding Candidate Effectors

RNA-Seq time course data from *B. napus* infected with *S. sclerotiorum* (Seifbarghi et al., 2017) were analyzed to determine the expression profiles of the 105 genes encoding *S. sclerotiorum* candidate effectors during different infection stages on *B. napus* leaves. Of these, the expression of 27 genes was up-regulated *in planta* during infection compared to *S. sclerotiorum* grown in culture. These effectors exhibited a wide range of expression patterns and were considered as the best candidates (**Table 1**). Eight genes were up-regulated at the very early stages of the infection (1 hpi), while eight genes were up-regulated at the later stages (48 hpi). Further annotation of the proteins using BLASTP revealed that many did not possess known structural domains (**Table 1**). Two genes, namely cutinase (SS1G_07661) (Bashi et al., 2012) and SsSSVP1 (SS1G_02068) (Lyu et al., 2016), had been investigated previously and were excluded from further analysis. An additional gene, SS1G_03611, was also excluded due to difficulties in cloning that was likely caused by mis-annotation arising from incorrect assembly of the original genome sequence.

**Table 1:** List of *Sclerotinia sclerotiorum* candidate effectors.

Gene ID Version I	Gene ID Version II^1^	Genbank Accession Number	Description^2^	Expression Level (hpi)^3^					
				1	3	6	12	24	48
**Necrosis-inducing effectors**									
SS1G_07027*^4^	Sscle06g052360	XP_001591581	hypothetical protein	10.1	2.9	3.8	4.0	3.1	8.8
SS1G_00872*	Sscle03g023810	XP_001598783	hypothetical protein	2.1	2.9	2.6	–	–	–
SS1G_00849*	Sscle03g024000	XP_001598760	22 kDa glycoprotein	7.4	3.2	–	–	–	7.1
SS1G_10096	Sscle16g107670	XP_001588549	SsCP1	3.8	3.7	–	–	–	3.3
SS1G_09232*	Sscle15g106560	XP_001589511	hypothetical protein	2.3	–	–	–	–	2.7
SS1G_08706*	Sscle14g098920	XP_001589942	plant expansin	–	–	–	3.1	4.6	4.8
SS1G_09150*	Sscle15g107190	XP_001589429	hypothetical protein	–	–	–	–	28.8	92.3
SS1G_11912	Sscle12g090490	XP_001586883	SsNEP2	–	–	–	–	5.8	8.2
SS1G_02068	Sscle01g003850	XP_001597872	SsSSVP1	–	–	–	–	–	21.6
**Non necrosis-inducing effectors**									
SS1G_07661	Sscle11g084380	XP_001591036	cutinase	3.0	3.9	4.3	4.7	3.4	–
SS1G_00263	Sscle03g028510	XP_001598177	SsV263	4.8	–	–	–	–	49.2
SS1G_04519	Sscle02g016170	XP_001594711	hypothetical protein	2.1	–	–	–	–	3.7
SS1G_09844	Sscle01g001890	XP_001589211	hypothetical protein	–	6.7	9.1	6.0	–	–
SS1G_13668	Sscle14g097630	XP_001585429	GAS2 domain-containing	–	7.4	6.8	5.3	–	–
SS1G_07669	Sscle11g084330	XP_001591044	DNase1 protein	–	6.9	3.6	3.4	–	–
SS1G_01867	Sscle01g005390	XP_001597671	long lifespan	–	2.5	–	–	–	–
SS1G_13126	Sscle02g020840	XP_001586033	hypothetical protein	–	–	25.9	13.6	10.3	12.5
SS1G_04382	Sscle02g017190	XP_001594575	thaumatin-like protein	–	–	–	–	4.8	8.2
SS1G_05939	Sscle05g046060	XP_001593017	hypothetical protein	–	–	–	–	31.8	–
SS1G_03611	Sscle07g055350	XP_001595522	cysteine-rich protein	–	–	–	–	85.1	247.0
SS1G_09420	Sscle15g105160	XP_001589698	hypothetical protein	–	–	–	–	–	6.0
SS1G_13696	Sscle14g097380	XP_001585457	hypothetical protein	–	–	–	–	–	8.3
SS1G_10534		XP_001588088	hypothetical protein	–	–	–	–	–	31.8
SS1G_09248	Sscle15g106410	XP_001589527	hydrophobin precursor	–	–	–	–	–	6.1
SS1G_00744	Sscle03g024730	XP_001598655	hypothetical protein	–	–	–	–	–	2.1
SS1G_01235	Sscle01g010360	XP_001597041	hypothetical protein		–	–	–	–	34.6
SS1G_02904		XP_001596682	SsCVNH	–	–	–	–		3.9

^1^Genome version I (Amselem et al., 2011) and version II models (Derbyshire et al., 2017).

^2^Annotation based on BLAST reports and gene symbols that have already been reported.

^3^Fold change relative to 0 h post-inoculation (hpi). (-) No significant change in expression.

^4^*Necrosis-inducing proteins identified in the current study.

### Identification of Cell Death Inducing Effectors by *In Planta* Transient Expression

**Figure 1:**
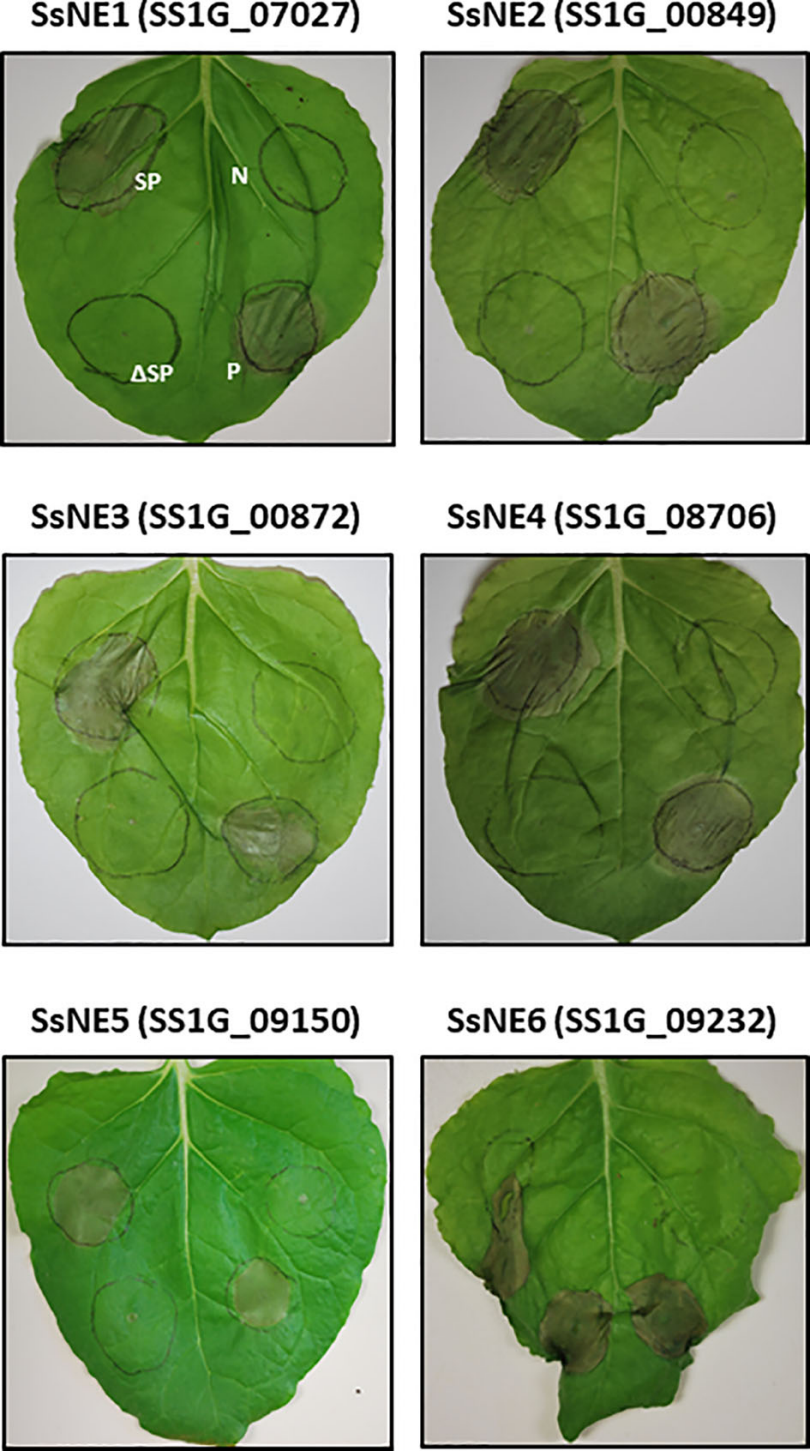
Necrosis-inducing activity of *Sclerotinia sclerotiorum* necrosis-inducing effectors using *Agrobacterium tumefaciens*-mediated transient expression in *Nicotiana benthamiana*. Leaves were infiltrated with *A. tumefaciens* harboring various constructs. Infiltrated zones on each leaf show constructs expressing *S. sclerotiorum* effectors with a signal peptide (SP), constructs expressing *S. sclerotiorum* effectors without a signal peptide (ΔSP), a construct expressing the empty vector as negative control (N), and a construct expressing the Nep1-like protein (PiNPP1.1) from *Phytophthora infestans* as positive control (P).

*In planta* transient expression was conducted to examine the necrotizing activity of the 24 candidate effectors (**Figure 1**). *A. tumefaciens* strains carrying constructs to express each protein with or without their SP, along with positive and negative controls, were infiltrated into *N. benthamiana* leaves. Six of the candidate proteins induced cell death. These effectors were named *S. sclerotiorum* Necrosis-inducing Effectors SsNE1 (SS1G_07027), SsNE2 (SS1G_00849), SsNE3 (SS1G_00872), SsNE4 (SS1G_08706), SsNE5 (SS1G_09150) and SsNE6 (SS1G_09232). Only one of these, SsNE6, displayed a necrosis phenotype both with and without the SP, while the others induced cell death only when the SP was included.

### Subcellular Localization of Necrosis-Inducing Effectors

**Figure 2:**
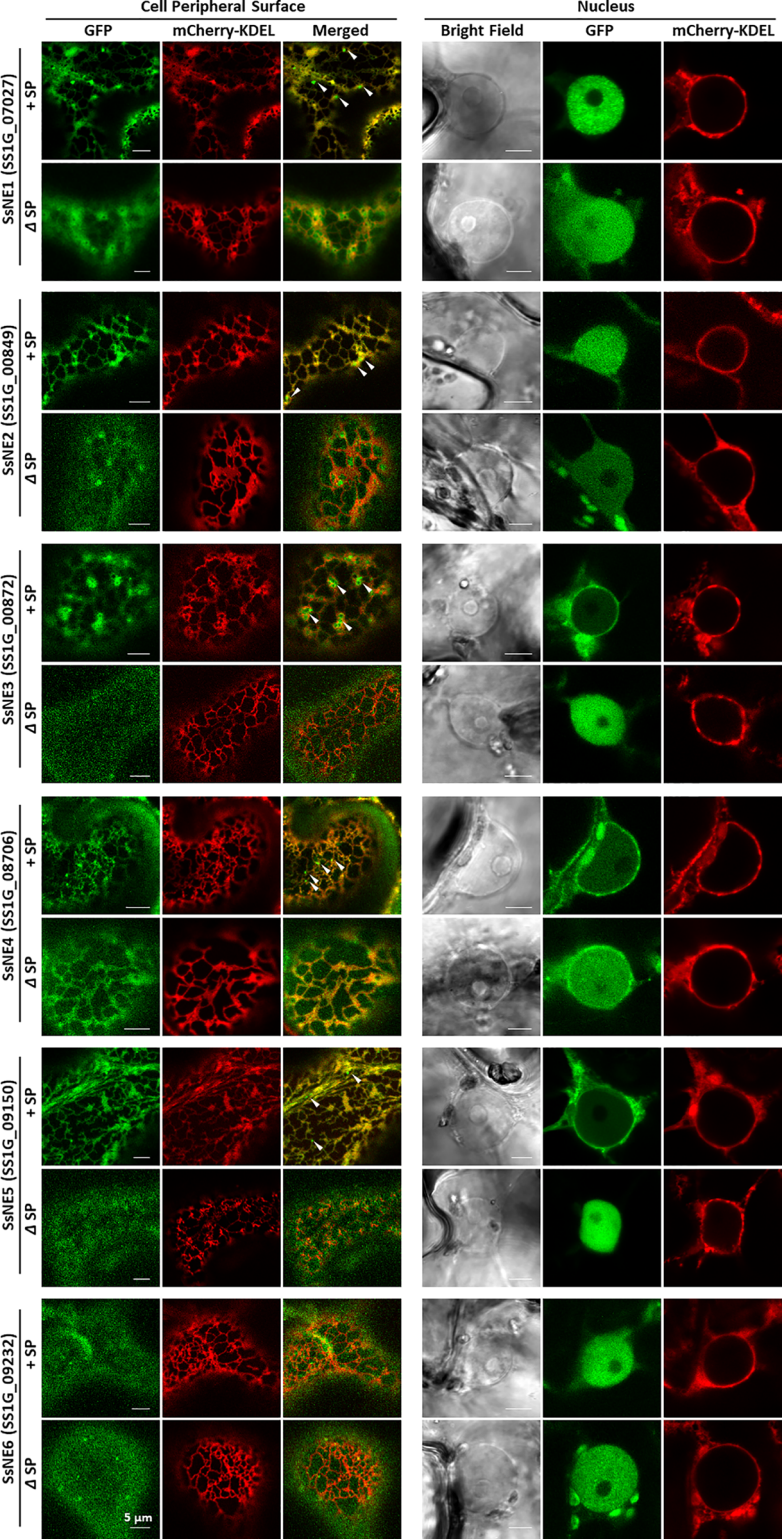
Subcellular localization of *Sclerotinia* *sclerotiorum* necrosis-inducing effectors in *Nicotiana benthamiana* leaves. Each effector protein was fused to GFP at the C-terminus and tested with (+SP) and without its native signal peptide (ΔSP). All constructs expressing effector proteins were co-infiltrated into leaves with a construct expressing an mCherry protein tagged with an ER retention signal (mCherry::KDEL). Punctate signals adjacent to ER tubules that were not labeled by the mCherry-ER lumen marker are indicated with arrows.

The subcellular localization of the six necrosis-inducing effectors was investigated by agro-expression of effectors with or without a SP and fused to a C-terminal GFP tag in *N. benthamiana* leaves (**Figure 2**; **Table S4**). Leaf discs collected at 36–48 h post-infiltration were observed under a confocal microscope. When full-length effector proteins with the endogenous SP were expressed, strong signals for SsNE1, SsNE2, and SsNE6 were detected in the nucleus, whereas SsNE3, SsNE4, and SsNE5 displayed intense signals on the peripheral nuclear surface and weakly within the nucleus. GFP signal for all six effector proteins without the SP appeared within the nucleus, but not within the nucleolus. In addition to association with the nucleus, five necrosis-inducing effector proteins (SsNE1–SsNE5) with a SP localized to the endomembrane network, while SsNE6 showed cytoplasmic localization. To confirm co-localization with the ER, effector proteins fused with GFP were simultaneously expressed with an mCherry-ER lumen marker (mCherry-KDEL). Confocal imaging revealed that all five proteins (SsNE1–SsNE5) with a SP displayed an overall co-localization with mCherry-KDEL, indicating that SsNE1–SsNE5 proteins associated with the ER. The SsNE1–SsNE5 proteins also produced additional punctate signals adjacent to the ER tubules that were not labeled by the mCherry-ER lumen marker. In contrast, SsNE1–SsNE5 proteins without a SP displayed a less cohesive ER-localization pattern (SsNE1, SsNE2, and SsNE4) or diffuse cytoplasmic localization (SsNE3 and SsNE5). SsNE6 proteins with or without a SP localized to the cytoplasm, revealing that this effector protein has a different cellular localization pattern compared to SsNE1–SsNE5. In summary, five necrosis-inducing effectors (SsNE1–SsNE5) exhibited similar cellular localization patterns and were primarily associated with ER-structures and the nucleus, suggesting a possible conserved mechanism of action.

### Targeting Effector Proteins to the Secretory Pathway Is Required to Trigger Cell Death

**Figure 3:**
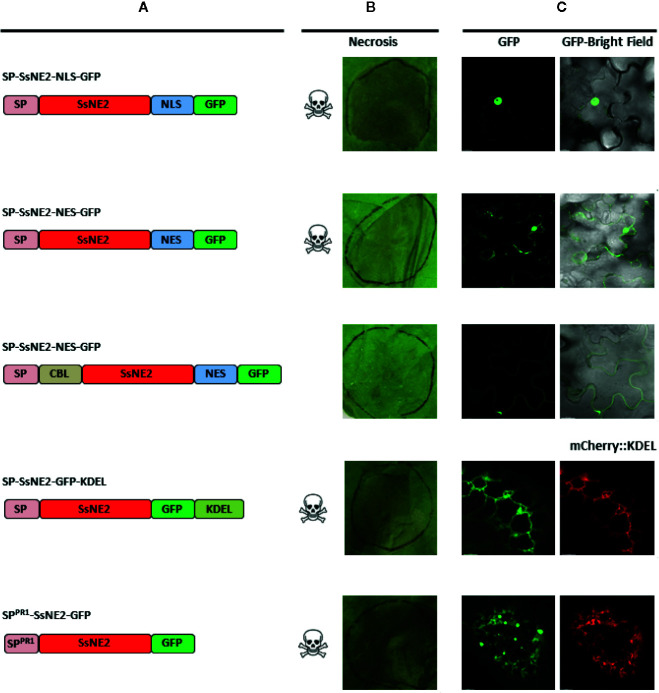
Subcellular localization and necrotizing activity of SsNE2 with its native SP and tagged with various targeting signals upon *Agrobacterium* *tumefaciens*-mediated expression in *Nicotiana benthamiana* leaves. **(A)** Schematic representation of the constructs showing native signal peptide (SP), signal peptide derived from *A. thaliana* Pathogenesis-related Protein 1 (SP^PR1^), SsNE2 protein (SsNE2), nuclear localization signal (NLS), nuclear export signal (NES), calcineurin B–like protein 1 myristoylation signal (CBL1), endoplasmic reticulum (ER) retention signal (KDEL) and green fluorescent protein (GFP). **(B)** Analysis of necrosis produced by each SnNE2 protein tagged with various targeting signals in *N. benthamiana* leaves. **(C)** Microscopic localization of each SsNE2 protein fused to GFP and tagged with various targeting signals. To confirm ER localization, constructs expressing effector proteins were co-infiltrated with a construct expressing an mCherry protein tagged with an ER retention signal (mCherry::KDEL).

The SsNE1–5 effectors were functional only when the SP was appended. This suggested that they are secreted to the extracellular space where they would naturally occur when delivered by the pathogen and encountered by the host. However, to determine if the localization to the ER/endomembrane system and/or the nucleus observed with the GFP fusions was involved in necrotizing activity, *A. tumefaciens* strains were constructed that expressed SsNE2 fused to various subcellular targeting signals. Constructs with or without the SP were generated; however, only those that included the SP induced necrosis. SsNE2 with a SP and fused to a NLS (SP-SsNE2-NLS-GFP) was detected mostly in the nucleus and induced cell death (**Figure 3**). SsNE2 with a SP and fused to a NES (SP-SsNE2-NES-GFP) was still detected in the nucleus, though to a lesser extent than in the absence of a NES, as well as in the cytosol and induced cell death. The C-terminal KDEL motif returns proteins to the ER from the Golgi apparatus and was fused to SsNE2 with a SP (SP-SsNE2-GFP-KDEL). This protein localized to the ER and was able to induce cell death, although it exhibited a delayed response and reduced incidence of necrosis in comparison with the native protein. The CBL1 motif is a PM targeting signal and prevents post-ER secretion to the extracellular space (Batistic et al., 2008). SsNE2 including a SP and fused to the CBL1 motif and NES motifs (CBL-SsNE2-NES-GFP) was exclusively detected in the PM; however, this protein did not induce cell death.

To confirm that targeting to the secretory pathway is required for cell death-inducing activity, the endogenous SP of SsNE2 was replaced with a plant SP from *N. tabacum* PR1. GFP signal from this protein (SP^PR1^-SsNE2-GFP) was detected in the ER (**Figure 3**). Treatment of infiltrated leaf tissues expressing SP^PR1^-SsNE2-GFP with 0.85 M KCL to induce plasmolysis did not reveal GFP signal in the apoplast, in contrast to PR1 linked to mCherry (data not shown). However, the amount of SsNE2-GFP protein in the apoplast may have been below the limits of detection *via* CLSM and GFP does not fluoresce well in the acidic environment of the apoplast. Expression of SP^PR1^-SsNE2-GFP induced cell death, indicating that both native fungal and heterologous plant SPs are functional. Taken together, all SsNE2 protein variants were capable of inducing necrosis when a SP was included, except for that tagged with the CBL1 motif which is shuttled to the PM. This confirmed that secretion to the extracellular space was required for necrotizing activity of SsNE2, and likely the other effector proteins whose activity is dependent upon the presence of a SP.

### Structural Topology of SsNE2 Proteins Necessary for Necrosis-Inducing Activity

**Figure 4:**
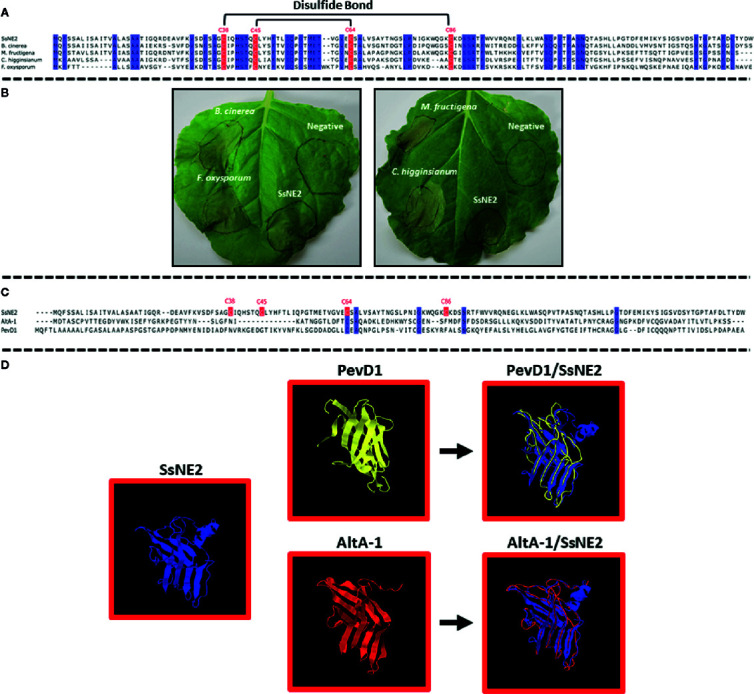
*S. sclerotiorum* SsNE2 and orthologous proteins in other pathogenic fungi. **(A)** Multiple sequence alignment of SsNE2 and its orthologs from *Botrytis cinerea* (BCIN_14g01200, GenBank accession XP_001552872.1), *Colletotrichum higginsianum* (CHEC91, GenBank accession XP_018152473.1), *Monilinia fructigena* (DID88_010138, GenBank accession RAL61042.1), and *Fusarium oxysporum* f.sp. *lycopersici* (FOXG_04016, GenBank accession XP_018238493.1) with prediction of disulfide bonds between the cysteine residues based on Predict Protein software. The conserved cysteine residues are marked with red boxes. Blue regions show high sequence identity. **(B)** Necrosis induced by *S. sclerotiorum* SsNE2 and orthologous proteins from other pathogenic fungi in *Nicotiana benthamiana* leaves after *Agrobacterium tumefaciens*-mediated transient expression. **(C)** Alignment of *S. sclerotiorum* SsNE2 and its structural homologues *Alternaria alternata* AltA-1 (GenBank KP275779) and *Verticillium dahliae* PevD1 (GenBank XP_009651683). The cysteine residues are marked with a red box. Blue regions show sequence identity. **(D)** Structures of *S. sclerotiorum* SsNE2, AltA-1 (PDB 3V0R) and PevD1 (PDB 5XMZ) with overlays of AltA-1 (red) and PevD1 (yellow) on SsNE2 (dark blue).

A BLASTP search with the six necrosis-inducing effectors identified putative orthologs in other necrotrophic and hemibiotrophic plant pathogens with sequence identities ranging from 40 to 90% (**Figure S1**, **Figure 4A**). Putative SsNE2 orthologs were identified in other ascomycete pathogens, such as *B. cinerea* (66% identity to BCIN_14g01200), *M. fructigena* (65.77% identity to DID88_010138), *C. higginsianum* (59.38% identity to CHEC91) and *F. oxysporum* f. sp. *lycopersici* (42.55% identity to FOXG_04016) (**Figure 4A**). All of these proteins induced necrosis when transiently expressed in *N. benthamiana* using the agro-expression system (**Figure 4B**). The necrosis symptoms induced by *M.* *fructigena* DID88_010138 were visible within 24–48 h post-infiltration, prior to the appearance of necrosis induced by SsNE2. The results revealed that the SsNE2 effector sequence and function is conserved within a wide range of phytopathogenic fungal species.

3D structure prediction of six necrosis-inducing effectors using SWISS-MODEL workspace (http://swissmodel.expasy.org/interactive) and I-TASSER (https://zhanglab.ccmb.med.umich.edu/I-TASSER/) showed that SsNE1, SsNE3, SsNE5, and SsNE6 did not have structural similarity to other proteins, while SsNE4 showed structural similarity with cerato-platanin and cerato-platanin-like proteins. Cerato-platanins are secreted by a wide variety of pathogenic fungi and possess a single domain with double ψβ-barrel with similarity to hydrophobins, expansins and glucanases (Gaderer et al., 2014) and are capable of inducing cell death (Scala et al., 2004; Fontana et al., 2008; Bernardi et al., 2011). The 3D structure prediction of SsNE2 showed that it was similar to the *Alternaria alternata* Major Allergen 1 (AltA-1), as reported previously by Guyon et al. (2014), and the PevD1 elicitor from *Verticillium dahliae* (**Figure 4D**) (Zhou et al., 2017; Zhang et al., 2019), although alignment of SsNE2 with these proteins revealed only a low level of sequence similarity (**Figure 4C**).

**Figure 5:**
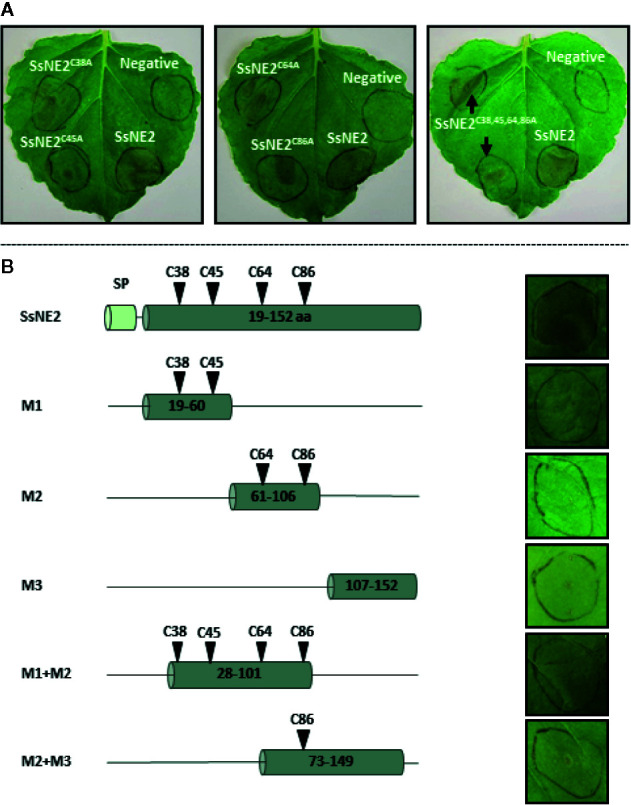
The effects of cysteine residue mutation and derived peptides from *Sclerotinia sclerotiorum* SsNE2 on the necrotizing activity. **(A)** SsNE2 cysteine residues at positions of C^38^, C^45^, C^64^, and C^86^ were either individually or simultaneously replaced by alanine and constructs infiltrated into *N. benthamiana* leaves using *A. tumefaciens*. Wild type SsNE2 used as a positive control and *A. tumefaciens* strains carrying an empty vector as a negative control (N). **(B)** Schematic representation of peptides derived from SsNE2 (left hand panels denoted as M1, M2, M3, M1+M2, and M2+M3) that were infiltrated into *N. benthamiana* leaves (right hand panels).

SsNE2 is a 152 amino acid protein with four cysteine residues (C^38^, C^45^, C^64^, and C^86^) that are conserved in the orthologs from other phytopathogenic fungi (**Figure S1**, **Figure 4A**). Conserved cysteine residues are likely to form internal disulfide bonds that are important for the protein structural integrity. Structural analysis of SsNE2 using Predict Protein (https://www.predictprotein.org/) predicted the formation of disulfide bonds between C^38^-C^86^ and C^45^-C^64^. To determine whether the necrotizing activity of SsNE2 depends on disulfide bonds between the cysteine residues, and thus an intact tertiary structure, C^38^, C^45^, C^64^, and C^86^ were replaced with alanine, either individually or simultaneously. All of the mutant constructs were transiently expressed using agro-expression in *N. benthamiana* leaves along with the wild type SsNE2 and the empty vector (AtEV) as a negative control. The incidence of necrotic symptoms was reduced by 40% in SsNE2^C38A^ and SsNE2^C45A^, by 50% in SsNE2^C64A^, by 60% in SsNE2^C86A^, and by 50% in SsNE2^C38A,C45A,C64A,C86A^ which lacked all four cysteine residues. The lesions that did form were also much smaller compared to the lesions caused by the wild type SsNE2 indicating that potency, but not activity has been compromised (**Figure 5A**). These results indicate that cysteine residues are important, but not absolutely critical, for the necrotizing activity of this protein.

To determine if a specific internal peptide sequence(s) of SsNE2 was required for necrosis-inducing activity, five truncated peptides were synthesized (**Figure 5B**). None of the peptides induced necrosis 7 days after infiltration in *N. benthamiana* leaves (**Figure 5B**). The SsNE2 protein is too large to be made synthetically; however, infiltration of *N. benthamiana* leaves with the culture supernatant from an *E. coli* strain expressing the entire SsNE2 protein (without signal peptide) resulted in necrosis symptoms 3 days post-infiltration (**Figure S2**). These results indicate that the full-length SsNE2, or a higher order structure, are required to induce cell death in host plants. Collectively, these results illustrate that a novel *S. sclerotiorum* effector protein, SsNE2, with evolutionary conserved orthologs among distantly-related phytopathogenic fungi possesses a unique structure involved in necrosis-inducing activity. As such, structural comparisons between candidate effectors from pathogenic fungi may allow identification of effector proteins that have little sequence similarity, but share similar functions.

### Receptor-Associated Kinases SOBIR1 and BAK1 Are Required for Necrotizing Activity of Effectors

**Figure 6:**
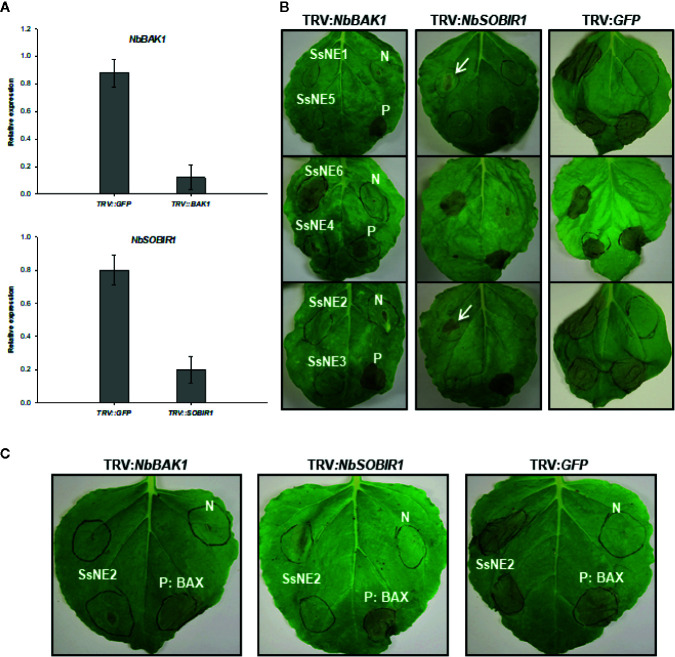
The effect of virus-induced gene silencing (VIGS) of *NbSOBIR1* and *NbBAK1* on the necrotizing activity of *Sclerotinia sclerotiorum* necrosis-inducing effectors. **(A)** *Nicotiana benthamiana* *SOBIR1* and *BAK1* expression after VIGS treatment as determined by qRT-PCR analysis. Expression is relative to *actin* as an endogenous control. Means and standard errors of three biological replicates are shown. **(B)** *N. benthamiana* plants were subjected to VIGS using the TRV-based vectors (*TRV : NbBAK1* and *TRV : NbSOBIR1*) and leaves infiltrated with *Agrobacterium tumefaciens* strains carrying effector constructs three weeks after TRV infiltration. *TRV : GFP* was used as a VIGS negative control. Bcl2-Associated protein X (BAX) was used as a BAK1/SOBIR1-independent positive control (P) for necrosis induction and *A. tumefaciens* carrying an empty vector as a negative control (N). Leaves in each row were infiltrated with the four constructs at the same locations as shown for the first leaf. Arrows show lesions that were smaller than the control plants. **(C)** The effect of *NbSOBIR1* and *NbBAK1* silencing on the necrotizing activity of recombinant SsNE2. *N. benthamiana* plants were subjected to VIGS using *TRV : NbBAK1* and *TRV : NbSOBIR1*. *TRV : GFP* was used as a VIGS negative control. Leaves were infiltrated with recombinant SsNE2 expressed in *Escherichia coli* three weeks after TRV infiltration. Bcl2-Associated protein X (BAX) was used as a BAK1/SOBIR1-independent positive control (P) for necrosis induction and *A. tumefaciens* carrying an empty vector as a negative control (N).

The dependency of necrotizing activity on the presence of a SP lends support to the notion that they must be directed to the plant’s secretory pathway(s) where they interact with their natural host target(s), possibly receptors. To test this hypothesis, the impact of silencing genes encoding the receptor-like kinases (RLKs) BAK1 and SOBIR1, which are known to be part of PAMP and ETI receptor complexes, was tested on the necrotizing activity of the six necrosis-inducing proteins. A tobacco rattle virus (TRV)-based virus induced gene silencing (VIGS) system was used. The gene silencing efficiency of the TRV‐VIGS system was monitored by VIGS of the phytoene desaturase (PDS) gene in *N. benthamiana* and resulted in photo‐bleaching of plant tissues two to three weeks after agro-expression both proximal and distal to the inoculation site indicating systemic spread of the virus. qRT-PCR analysis revealed that *NbSOBIR1-* and *NbBAK1-*silenced plants had 80% lower *SOBIR1* and *BAK1* transcript levels compared to the control *GFP-*silenced plants (**Figure 6A**). In *NbSOBIR1-* and *NbBAK1-*silenced plants, the five necrosis-inducing proteins (SsNE1–SsNE5) with a requirement for secretion exhibited reduced incidence and/or reduced necrosis symptoms compared with the control plants (**Figure 6B**). For SsNE2, necrosis was observed in 55% and 43% of *NbSOBIR1*- or *NbBAK1*- silenced plants, respectively, compared with 89% in the control plants. SsNE1 generated necrosis symptoms in 50% and 42% of *NbSOBIR1-* or *NbBAK1-* silenced plants, respectively, compared with 91% in the control plants. Necrosis was observed in 40% and 35% with SsNE4, in 35% and 30% with SsNE5, and in 42% and 50% with SsNE3 of the *NbSOBIR1-* or *NbBAK1-*silenced plants, respectively, compared with 90% in the control plants. The lesions that did form were also much smaller (**Figure 6B**) and the necrosis symptoms delayed by 2 to 3 days in the silenced plants compared with the control plants. The necrotizing activity of SsNE6, which does not require a SP for its activity, was independent of BAK1 and SOBIR1, as their silencing did not change the incidence or severity of necrosis caused by this effector compared to the control plants. Additionally, expression of BAX (proapoptotic factor Bcl2-Associated protein X) triggered strong necrosis in both the control and *NbSOBIR1-* or *NbBAK1-*silenced plants, which is in line with the fact that BAX causes plant programmed cell death in a BAK1/SOBIR1-independent manner (Liebrand et al., 2013). Together, these results demonstrated that BAK1 and SOBIR1 are required for necrosis triggered by the extracellular SsNE1–SsNE5 effectors, whereas necrosis caused by SsNE6, which is active when present in the cytosol, is independent of BAK1 and SOBIR1.

To further confirm the involvement of these two RLKs in the induction of necrosis caused by SsNE2, leaves of the control and *NbSOBIR1-* and *NbBAK1-*silenced *N. benthamiana* plants were infiltrated with recombinant SsNE2 expressed in *E. coli*. Infiltration with SsNE2 caused strong necrosis in control plants, whereas the incidence and severity of necrosis in both *NbSOBIR1-* and *NbBAK1-*silenced plants were much lower (**Figure 6C**). These results suggest that SsNE2 proteins act in the extracellular space where they are perceived by a BAK1 and SOBIR1-dependent receptor complex to initiate the cell death pathway.

## Discussion

In the current study, bioinformatic and transcriptomic approaches were combined to predict genes encoding putative effectors within the *S. sclerotiorum* genome. Of the 24 predicted effectors, 6 were found to exhibit necrotizing activity in *N. benthamiana*. The genes encoding four (SsNE1, SsNE2, SsNE3, and SsNE6) of the six necrosis-inducing proteins were expressed at the early stages of *S. sclerotiorum* infection (1 hpi) when the pathogen is just beginning to penetrate the host (Liang and Rollins, 2018). This suggests that *S. sclerotiorum* is secreting effectors at the very earliest stages of the infection to promote cell death. This likely creates regions of dead tissue allowing the pathogen to establish a foothold similar to that caused by the *P. tritici-repentis *necrosis-inducing effectors PtrToxA, PtrToxB, and PtrToxC. PtrToxA is a fibronectin type III-like peptide with an arginyl-glycyl-aspartic acid motif that interacts with Tsn1, a nucleotide-binding site leucine-rich repeat (NBS-LRR) domain and a serine/threonine protein kinase (S/TPK) domain protein. PtrToxB is a cysteine rich, apoplastic toxin, that interacts with the product of the *Tsc2* gene in ToxB-sensitive wheat cultivars (Ciuffetti et al., 2010; Figueroa et al., 2015; Corsi et al., 2020). Several of the candidate effectors identified in the present study were reported by Guyon et al. (2014), including SsNE2, SS1G_02904, SS1G_11912 (SsNep2) (Bashi et al., 2010) and SS1G_10096 (SsCP1) (Yang et al., 2018a).

SsNep2 and SsCP1 are known to induce cell death; however, they did not exhibit necrosis-inducing activity using the protocol in the current study. In the current study, transient expression using *A. tumefaciens* strains harboring vectors that use the CaMV *35S* promoter was used to deliver the effector proteins, whereas, the two previous studies (Bashi et al., 2010; Yang et al., 2018a) used virus-based transient expression systems. Kettles et al. (2017) also reported variation between experiments using *A. tumefaciens* versus a virus-based system in testing *Z. tritici* effectors for necrotizing activity in *N. benthamiana*. The necrosis was usually weak or sometimes absent with the *A. tumefaciens* system and was attributed to lower levels of expression in plants relative to the virus-based systems. The necrotizing activity of the six *S. sclerotiorum* effectors was comparable to *P. infestans* PiNPP1.1, except that the necrotic lesions were delayed by one or two days relative to PiNPP1.1. This suggests that the protocol used in the current study was reliable for identifying potent *S. sclerotiorum* necrosis-inducing effectors, while a virus-based system might identify these as well as other less potent necrosis-inducing effectors.

Five of the SsNE proteins were dependent on the presence of a SP for activity, but were localized to the ER/endomembrane system and/or the nucleus. CLSM did not detect GFP-tagged SsNE proteins in the apoplast after plasmolysis; however, it should be noted that the acidic environment of the apoplast reduces GFP signal and a different fluorophore, such red fluorescent protein, might be better suited for such experiments. Nonetheless, these observations suggest that the necrosis-inducing proteins are secreted into the apoplast where they may interact with host targets. SsSSVP1 (SS1G_02068), a candidate effector identified in this study, also localizes to the ER when its native SP is intact (Lyu et al., 2016). To assess if localization to the ER/endomembrane system and nucleus was associated with necrotizing activity, SsNE2 was fused to various subcellular targeting signals. Despite various subcellular localizations mediated by these targeting signals, cell death was only observed when the SP was included. The NLS motif targets proteins to the nucleus, while the NES targets them to the cytoplasm (Kalderon et al., 1984); however, SsNE2 proteins fused to either still induced cell death. The NES didn’t completely translocate SsNE2 from the nucleus to the cytoplasm, but did reduce its nuclear concentration as was also observed with NES-tagged Avr2 from *F. oxysporum* (Ma et al., 2013). Using NLS-tagged Avr2, Ma et al. (2013) showed that nuclear localization of Avr2 is necessary for induction of R gene (I-2)-dependent cell death. Proteins with KDEL motifs are retrieved from downstream compartments of the secretory pathway and returned to the ER (Stornaiuolo et al., 2003). SsNE2 with a KDEL motif induced necrosis, but the symptoms were delayed and the incidence of necrosis induction was reduced. Is has been reported that retrieval back to the ER from downstream compartments conferred by KDEL is not absolute, resulting in leakage of some protein into the secretory system (Stornaiuolo et al., 2003). Therefore, it is possible that a small amount of SsNE2-GFP-KDEL was deposited in the extracellular space and this might lead to the delayed response and reduced incidence of necrosis induction. To explore this possibility and to clarify whether the ER was involved in necrotizing activity, a CBL1 motif was incorporated into SsNE2. Proteins with a CBL1 motif are initially targeted to the ER where they undergo N-myristoylation; *S*-acylation then causes trafficking of the protein to the PM independent of the Golgi apparatus and by-passing the secretory pathway (Batistic et al., 2008). SsNE2-GFP with a CBL1 motif was localized to the PM and was unable to induce cell death. Furthermore, recombinant SsNE2 was able to induce necrosis when applied directly to plant tissues. These results provide good evidence that SsNE2, and likely SsNE1, SsNE3, SsNE4, and SsNE5, must be present, at least initially, in the extracellular space to induce necrosis.

Effectors secreted by pathogens are often small, cysteine-rich proteins and the disulfide bonds formed between the cysteine residues are important for their stability in the extra-cellular environment (Rep, 2005; Kamoun, 2006). Disulfide bonds are important for the activity of the apoplastic effector PtrToxB (Figueroa et al., 2015). The necrosis-inducing effectors identified in the current study were small, secreted cysteine-rich proteins with four cysteine residues in SsNE2, SsNE4, SsNE5, and SsNE6, and six in SsNE1 and SsNE3. The four cysteine residues in SsNE2 appear to play a role in the necrotizing activity as substitution with alanine reduced the incidence and severity of necrosis. The C^64^ and C^86^ residues and adjacent regions were conserved between SsNE2 and orthologs in phylogenetically distant pathogens. C^86^ mutation showed the highest degree of symptom reduction compared to the others; however, the cysteine mutations, either individually or simultaneously, did not completely abolish necrotizing activity indicating that they are not absolutely required for activity. They may, however, still be important for structural integrity and long-term stability of this protein to maximize necrotizing activity or in presenting an epitope to plant receptors in an optimal configuration. SsNE2 peptides spanning the protein were unable to induce necrosis indicating that smaller, independent motifs that might be responsible for induction of necrosis were not present. Similarly, NLP proteins possess a conserved heptapeptide motif (GHRHDWE) that is essential for necrosis-inducing activity; however, this peptide alone does not cause necrosis suggesting that the 3D structure of NLP proteins might be important for interaction of the peptide epitope with targets in the host plant (Schouten et al., 2008; Cabral et al., 2012; Böhm et al., 2014; Albert et al., 2015). This is in contrast to *B. cinerea* BcXyl1 where a 26-amino acid peptide is capable of inducing cell death (Yang et al., 2018b). The partially impaired function of SsNE2 with mutated cysteine residues could also indicate that the cysteine residues might not form disulfide bonds. Binding to metal ions, such as Fe^2+/3+^, Zn^2+^, Cd^2+^, and Cu^+^, and redox activity are additional properties associated with cysteine residues (Giles et al., 2003). The partially impaired function of SsNE2 variants mutated in cysteine residues could also be associated with reduced metal-binding that may impair interaction with a cognate host target.

Orthologs of SsNE2 in four other fungal pathogens, including *B. cinerea*, *F. oxysporum*, *C. higginsianum* and *M. fructigena*, also possessed necrosis-inducing activity. Previously, Guyon et al. (2014) reported that the small, secreted protein encoded by SS1G_00849, denoted here as SsNE2, was similar to the *A. alternate* AltA-1 allergen and the *C. hingginsianum* CHEC91 effector. Our 3D structure prediction of SsNE2 also showed that it was highly similar to AltA-1, as well as PevD1 from *Verticillium dahliae* (Zhou et al., 2017; Zhang et al., 2019), although alignment of SsNE2 with these proteins revealed only a low level of sequence similarity. AltA-1-like proteins (AA1s; Pfam family PF16541) have been found in the Dothideomycetes and Sordariomycetes fungal classes (Chruszcz et al., 2012); however, their discovery in *S. sclerotiorum* would extend this to Leotiomyces as well. Despite their wide distribution, little is known about the contribution of AltA-1-like proteins to fungal pathogenicity and they appear to have a variety of roles. PevD1 induces necrosis when transiently expressed in *N. benthamiana* in a SP-dependent manner (Zhang et al., 2019) similar to SsNE2; however, it also interferes with the function of Pathogen-Related Protein 5 (Zhang et al., 2019) and indirectly induces early flowering in plants infected with *V. dahliae* through interaction with NRP, an asparagine-rich protein that regulates Cryptochrome 2 localization (Zhou et al., 2017). Interestingly, *C. hingginsianum* *CHEC91* is not among the wave of effector genes expressed during the switch to the necrotrophic phase, but is primarily expressed when the pathogen is grown on artificial media (Kleemann et al., 2012), a saprophytic-like environment that would exist in the host only after tissue necrosis has occurred. This underlines the importance of structural comparisons to elucidate conserved functions among effectors with low levels of sequence similarity.

The dependency on secretion for activity may also indicate that the necrosis-inducing proteins interact with a target receptor(s) in the extracellular space leading to induction of cell death. In the wheat-pathogen *Zymoseptoria tritici*, most necrosis-inducing effectors need to be secreted to extracellular space to function (Kettles et al., 2017) and necrosis-inducing effectors from the rice false smut pathogen, *Ustilaginoidea virens*, also require a SP for full activity (Fang et al., 2016). Plant cells are equipped with cell surface PRRs that sense pathogen-derived components and subsequently activate downstream signal transduction pathways leading to induction of defense responses. PRRs participate in multi-protein complexes at the PM (Monaghan and Zipfel, 2012). RLPs are PRRs that lack a kinase domain and are dependent on other PRRs with a kinase domain to activate the downstream defense signal transduction pathway (Rivas and Thomas, 2005). SOBIR1 is one of the key regulatory proteins interacting with RLPs involved in triggering immunity (Liebrand et al., 2013; Zhang et al., 2013). BAK1 acts as either a co-receptor or a general regulator of downstream signaling pathways of the RLP complex (Monaghan and Zipfel, 2012; Liebrand et al., 2014). For example, the RLP23-BAK1-SOBIR1 complex is involved in the activation of plant immunity upon perception of *S. sclerotiorum* nlp20 (the conserved 20 amino acid peptide present in NLPs) (Albert et al., 2015). The RLP30-BAK1-SOBIR1 complex is required for induction of PTI in *A. thaliana* against *S. sclerotiorum* upon perception of SCFE1, an elicitor secreted by *S. sclerotiorum* (Zhang et al., 2013). Recognition of the AvrLm1 effector of the apoplastic fungus, *Leptosphaeria maculans*, by the *B. napus* RLP, LepR3, also requires SOBIR1 and BAK1 (Ma and Borhan, 2015). In the current study, VIGS of *NbBAK1* and *NbSOBIR1* lowered the incidence and severity of the necrosis phenotype for all of the necrosis-inducing proteins that were dependent upon secretion (SsNE1–SsNE5) lending support to the notion that the necrotizing activity for some effectors is dependent on SOBIR1 and BAK1. In support of this notion, *B. cinerea* BcXYG1, a xyloglucanase with necrosis-inducing activity, loses its necrosis-inducing activity when expressed in *bak1* or *sobir1* mutant plants (Zhu et al., 2017). Recognition of *B. cinerea* endopolygalacturonase also occurs *via* an RLP42 (Responsiveness to Botrytis polygalacturonase 1; RBPG1):SOBIR1 complex and necrosis is not induced in *sobir1* mutants (Zhang et al., 2014). Similarly, the necrotizing activity of *Z. tritici* effectors is dependent on a SOBIR1/BAK1-dependent pathway (Kettles et al., 2017). The necrotizing activity of SsNE6 was not dependent on BAK1 and SOBIR1. This was not surprising since, in contrast to other five necrosis-inducing effectors, SsNE6 does not require a SP for its necrotizing activity and was located in the cytosol. It is likely that this protein targets a cytoplasmic protein leading to induction of cell death.

The discoveries made through this study have led to the identification of six novel necrosis-inducing effectors from *S. sclerotiorum*, which might facilitate colonization of host tissues during the infection. *S. sclerotiorum* is known for its broad host range, suggesting that its effectors might be host non-specific and interact with RLPs that are common and widely distributed, especially in dicotyledonous plants. Alternatively, the availability of numerous necrosis-inducing effectors in *S. sclerotiorum* that are specifically recognized by different hosts might lead to susceptibility of a large number of plant species. The identification of host targets for these effectors may reveal factors conferring susceptibility to this pathogen and enable the development of strategies for enhancing *S. sclerotiorum* resistance. These effectors could be used for developing effector-guided breeding protocols for rapidly phenotyping germplasm.

## Data Availability Statement

All datasets generated for this study are included in the article/**Supplementary Material**.

## Author Contributions

SS, DH, YW, LM, and HM designed the study. SS performed the experiments. CC and DB provided technical assistance. SS wrote the manuscript. DH, YW, and HM revised the manuscript. All authors contributed to the article and approved the submitted version.

## Funding

This study was funded by SaskCanola and the Government of Canada through the Developing Innovative Agri-Products program.

## Conflict of Interest

The authors declare that the research was conducted in the absence of any commercial or financial relationships that could be construed as a potential conflict of interest.
