# Supplementary material for: Receptor-Like Kinases BAK1 and SOBIR1 Are Required for Necrotizing Activity of a Novel Group of Sclerotinia sclerotiorum Necrosis-Inducing Effectors
Source: Front Plant Sci. 2020 Jul 10;11:1021. doi: 10.3389/fpls.2020.01021 (PMC7367142; doi:10.3389/fpls.2020.01021)
Supplement: Supplementary file 2 [file Table_2.docx]

Table S2. Synthetic DNA sequences of SsNE2 tagged with various targeting signals*.

**Construct Name DNA Sequence**

| **SP-SsNE2-NLS** | GGGG**ACAAGTTTGTACAAAAAAGCAGGCTTC**ATGCAATTCTCATCTGCTCTTATCTCTGCCATCACCGTTGCTCTTGCTTCGGCTGCCACCATTGGACAACGTGACGAGGCCGTCTTCAAGGTCTCTGACTTCAGCGCAGGCTGTATCCAACACAGCACCCAGTGCCTCTACCACTTCACTCTCATCCAACCCGGTACCATGGAGACCGTCGGTGTTGAATGTTCCGCCCTCGTTAGCGCCTACACCAATGGCTCCCTCCCCAACATCGGCAAATGGCAAGGCAAATGCAAGGATTCTTCCCGCACTTTCTGGGTTGTCCGTCAAAACGAGGGTCTTAAGCTCTGGGCTTCTCAACCAGTTACTCCAGCTAGCAACCAAACCGCATCCCACCTCCTCCCAGGCACTGACTTTGAAATGATCAAATACTCTATCGGTAGCGTTGACAGCTACACTGGACCAACTGCTTTCGACTTGACCTACGACTGGCCTAAGAAGAAGAGAAAGGTT**GACCCAGCTTTCTTGTACAAAGTGGT**CCCC |
| --- | --- |
| **SP-SsNE2-NES** | GGGG**ACAAGTTTGTACAAAAAAGCAGGCTTC**ATGCAATTCTCATCTGCTCTTATCTCTGCCATCACCGTTGCTCTTGCTTCGGCTGCCACCATTGGACAACGTGACGAGGCCGTCTTCAAGGTCTCTGACTTCAGCGCAGGCTGTATCCAACACAGCACCCAGTGCCTCTACCACTTCACTCTCATCCAACCCGGTACCATGGAGACCGTCGGTGTTGAATGTTCCGCCCTCGTTAGCGCCTACACCAATGGCTCCCTCCCCAACATCGGCAAATGGCAAGGCAAATGCAAGGATTCTTCCCGCACTTTCTGGGTTGTCCGTCAAAACGAGGGTCTTAAGCTCTGGGCTTCTCAACCAGTTACTCCAGCTAGCAACCAAACCGCATCCCACCTCCTCCCAGGCACTGACTTTGAAATGATCAAATACTCTATCGGTAGCGTTGACAGCTACACTGGACCAACTGCTTTCGACTTGACCTACGACTGGAACGAGCTTGCTCTTAAGTTGGCTGGACTTGATATT**GACCCAGCTTTCTTGTACAAAGTGGT**CCCC |
| **SP-CBL-SsNE2^∆SP^-NES** | GGGG**ACAAGTTTGTACAAAAAAGCAGGCTTC**ATGGGCTGCTTCCACTCAAAGGCAGCAAAAGAATTTGCCACCATTGGACAACGTGACGAGGCCGTCTTCAAGGTCTCTGACTTCAGCGCAGGCTGTATCCAACACAGCACCCAGTGCCTCTACCACTTCACTCTCATCCAACCCGGTACCATGGAGACCGTCGGTGTTGAATGTTCCGCCCTCGTTAGCGCCTACACCAATGGCTCCCTCCCCAACATCGGCAAATGGCAAGGCAAATGCAAGGATTCTTCCCGCACTTTCTGGGTTGTCCGTCAAAACGAGGGTCTTAAGCTCTGGGCTTCTCAACCAGTTACTCCAGCTAGCAACCAAACCGCATCCCACCTCCTCCCAGGCACTGACTTTGAAATGATCAAATACTCTATCGGTAGCGTTGACAGCTACACTGGACCAACTGCTTTCGACTTGACCTACGACTGGAACGAGCTTGCTCTTAAGTTGGCTGGACTTGATATT**GACCCAGCTTTCTTGTACAAAGTGGT**CCCC |
| **SP-CBL-SsNE2-NES** | GGGG**ACAAGTTTGTACAAAAAAGCAGGCTTC**ATGCAATTCTCATCTGCTCTTATCTCTGCCATCACCGTTGCTCTTGCTTCGGCTGGCTGCTTCCACTCAAAGGCAGCAAAAGAATTTGCCACCATTGGACAACGTGACGAGGCCGTCTTCAAGGTCTCTGACTTCAGCGCAGGCTGTATCCAACACAGCACCCAGTGCCTCTACCACTTCACTCTCATCCAACCCGGTACCATGGAGACCGTCGGTGTTGAATGTTCCGCCCTCGTTAGCGCCTACACCAATGGCTCCCTCCCCAACATCGGCAAATGGCAAGGCAAATGCAAGGATTCTTCCCGCACTTTCTGGGTTGTCCGTCAAAACGAGGGTCTTAAGCTCTGGGCTTCTCAACCAGTTACTCCAGCTAGCAACCAAACCGCATCCCACCTCCTCCCAGGCACTGACTTTGAAATGATCAAATACTCTATCGGTAGCGTTGACAGCTACACTGGACCAACTGCTTTCGACTTGACCTACGACTGGAACGAGCTTGCTCTTAAGTTGGCTGGACTTGATATT**GACCCAGCTTTCTTGTACAAAGTGGT**CCCC |
| **SP-SsNE2-GFP-KDEL** | GGGG**ACAAGTTTGTACAAAAAAGCAGGCTTC**ATGCAATTCTCATCTGCTCTTATCTCTGCCATCACCGTTGCTCTTGCTTCGGCTGCCACCATTGGACAACGTGACGAGGCCGTCTTCAAGGTCTCTGACTTCAGCGCAGGCTGTATCCAACACAGCACCCAGTGCCTCTACCACTTCACTCTCATCCAACCCGGTACCATGGAGACCGTCGGTGTTGAATGTTCCGCCCTCGTTAGCGCCTACACCAATGGCTCCCTCCCCAACATCGGCAAATGGCAAGGCAAATGCAAGGATTCTTCCCGCACTTTCTGGGTTGTCCGTCAAAACGAGGGTCTTAAGCTCTGGGCTTCTCAACCAGTTACTCCAGCTAGCAACCAAACCGCATCCCACCTCCTCCCAGGCACTGACTTTGAAATGATCAAATACTCTATCGGTAGCGTTGACAGCTACACTGGACCAACTGCTTTCGACTTGACCTACGACTGGAGTAAAGGAGAAGAACTTTTCACTGGAGTTGTCCCAATTCTTGTTGAATTAGATGGTGATGTTAATGGGCACAAATTTTCTGTCAGTGGAGAGGGTGAAGGTGATGCAACATACGGAAAACTTACCCTTAAATTTATTTGCACTACTGGAAAACTACCTGTTCCGTGGCCAACACTTGTCACTACTTTCTCTTATGGTGTTCAATGCTTTTCAAGATACCCAGATCATATGAAGCGGCACGACTTCTTCAAGAGCGCCATGCCTGAGGGATACGTGCAGGAGAGGACCATCTTCTTCAAGGACGACGGGAACTACAAGACACGTGCTGAAGTCAAGTTTGAGGGAGACACCCTCGTCAACAGGATCGAGCTTAAGGGAATCGATTTCAAGGAGGACGGAAACATCCTCGGCCACAAGTTGGAATACAACTACAACTCCCACAACGTATACATCATGGCCGACAAGCAAAAGAACGGCATCAAAGCCAACTTCAAGACCCGCCACAACATCGAAGACGGCGGCGTGCAACTCGCTGATCATTATCAACAAAATACTCCAATTGGCGATGGCCCTGTCCTTTTACCAGACAACCATTACCTGTCCACACAATCTGCCCTTTCGAAAGATCCCAACGAAAAGAGAGACCACATGGTCCTTCTTGAGTTTGTAACAGCTGCTGGGATTACACATGGCATGGATGAACTATACAAAAAAGATGAGTTGTGA**GACCCAGCTTTCTTGTACAAAGTGGT**CCCC |
| **SP^PR1^-SsNE2** | GGGG**ACAAGTTTGTACAAAAAAGCAGGCTTC**ATGGGATTTGTTCTCTTTTCACAATTGCCTTCATTTCTTCTTGTCTCTACACTTCTCTTATTCCTAGTAATATCCCACTCTTGCCGTGCCGCCACCATTGGACAACGTGACGAGGCCGTCTTCAAGGTCTCTGACTTCAGCGCAGGCTGTATCCAACACAGCACCCAGTGCCTCTACCACTTCACTCTCATCCAACCCGGTACCATGGAGACCGTCGGTGTTGAATGTTCCGCCCTCGTTAGCGCCTACACCAATGGCTCCCTCCCCAACATCGGCAAATGGCAAGGCAAATGCAAGGATTCTTCCCGCACTTTCTGGGTTGTCCGTCAAAACGAGGGTCTTAAGCTCTGGGCTTCTCAACCAGTTACTCCAGCTAGCAACCAAACCGCATCCCACCTCCTCCCAGGCACTGACTTTGAAATGATCAAATACTCTATCGGTAGCGTTGACAGCTACACTGGACCAACTGCTTTCGACTTGACCTACGACTGG**GACCCAGCTTTCTTGTACAAAGTGGT**CCCC |

* AttB site sequences are denoted in bold font. Various targeting signals in each construct are marked in gray.
