# Supplementary material for: Receptor-Like Kinases BAK1 and SOBIR1 Are Required for Necrotizing Activity of a Novel Group of Sclerotinia sclerotiorum Necrosis-Inducing Effectors
Source: Front Plant Sci. 2020 Jul 10;11:1021. doi: 10.3389/fpls.2020.01021 (PMC7367142; doi:10.3389/fpls.2020.01021)
Supplement: Supplementary file 3 [file Table_3.docx]

Table S3. Amino acid sequences of peptides derived from SsNE2.

| **Peptide Name** | **Peptide Sequence** |
| --- | --- |
| M1 | ATIGQRDEAVFKVSDFSAGCIQHSTQCLYHFTLIQPGTMETV |
| M2 | GVECSALVSAYTNGSLPNIGKWQGKCKDSSRTFWVVRQNEGLKLWA |
| M2 | GVECSALVSAYTNGSLPNIGKWQGKCKDSSRTFWVVRQNEGLKLWA |
| M1+M2 | VFKVSDFSAGCIQHSTQCLYHFTLIQPGTMETVGVECSALVSAYTNGSLPNIGKWQGKCKDSSRTFWVVRQNEG |
| M2+M3 | NGSLPNIGKWQGKCKDSSRTFWVVRQNEGLKLWASQPVTPASNQTASHLLPGTDFEMIKYSIGSVDSYTGPTAFDL |
