# Supplementary material for: Receptor-Like Kinases BAK1 and SOBIR1 Are Required for Necrotizing Activity of a Novel Group of Sclerotinia sclerotiorum Necrosis-Inducing Effectors
Source: Front Plant Sci. 2020 Jul 10;11:1021. doi: 10.3389/fpls.2020.01021 (PMC7367142; doi:10.3389/fpls.2020.01021)
Supplement: Supplementary file 4 [file Table_4.docx]

**Table S4.** Subcellular localization of *S. sclerotiorum* necrosis-inducing effectors with and without a signal peptide (SP).

| **Gene ID** | **Protein Name** | **Subcellular localization** | |
| --- | --- | --- | --- |
|  |  | **with SP** | **without SP** |
| SS1G_09232 | SsNE6 | Cytoplasm + Nucleus | Cytoplasm + Nucleus |
| SS1G_00872 | SsNE3 | ER + Nucleus |  |
| SS1G_07027 | SsNE1 |  |  |
| SS1G_00849 | SsNE2 |  |  |
| SS1G_08706 | SsNE4 |  | ER + Nucleus |
| SS1G_09150 | SsNE5 |  | Nucleus |
