# Supplementary figures and images for: Receptor-Like Kinases BAK1 and SOBIR1 Are Required for Necrotizing Activity of a Novel Group of Sclerotinia sclerotiorum Necrosis-Inducing Effectors
Source: Front Plant Sci. 2020 Jul 10;11:1021. doi: 10.3389/fpls.2020.01021 (PMC7367142; doi:10.3389/fpls.2020.01021)

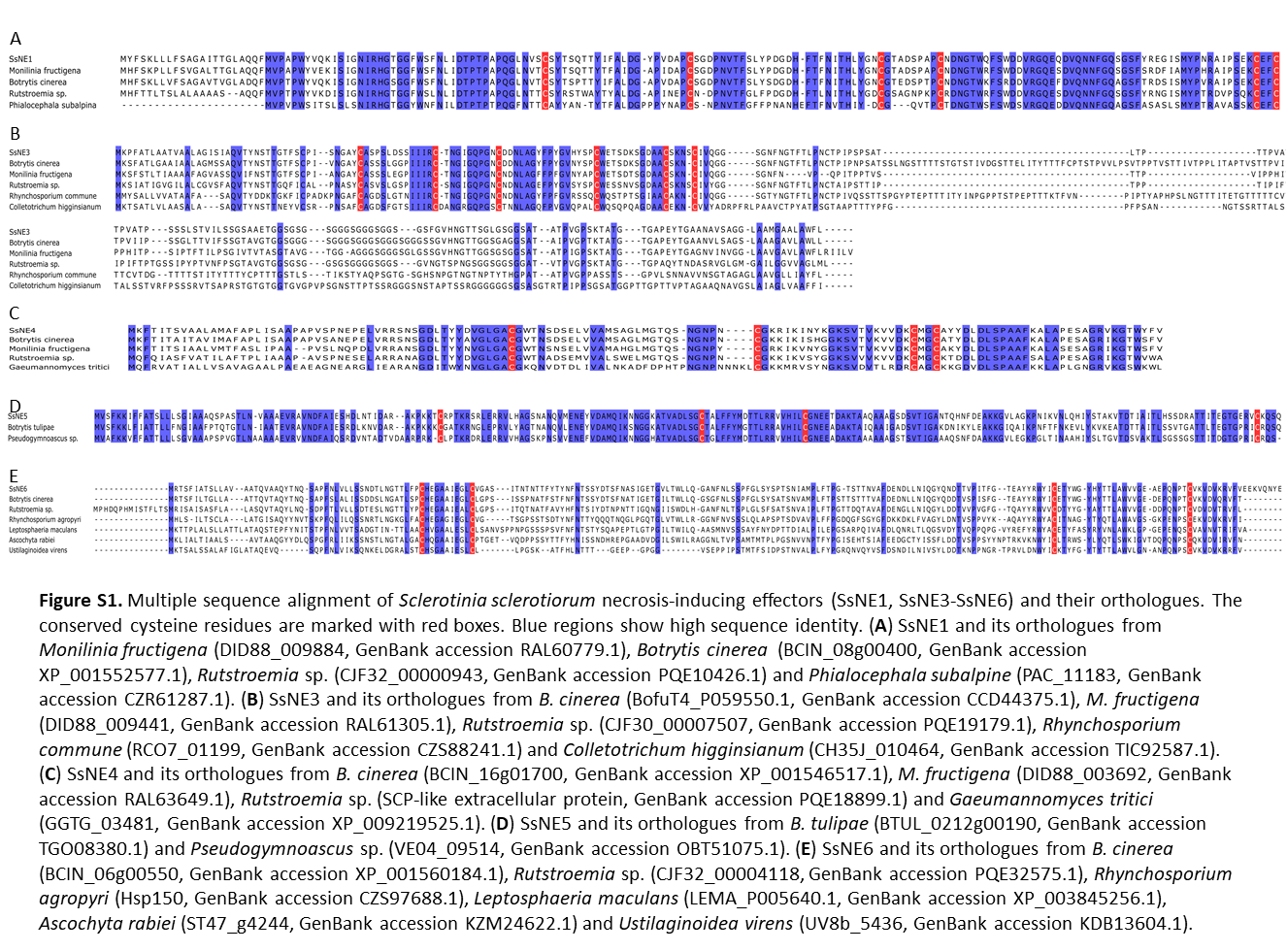

Supplement: Supplementary file 5 [file Image_1.tif]

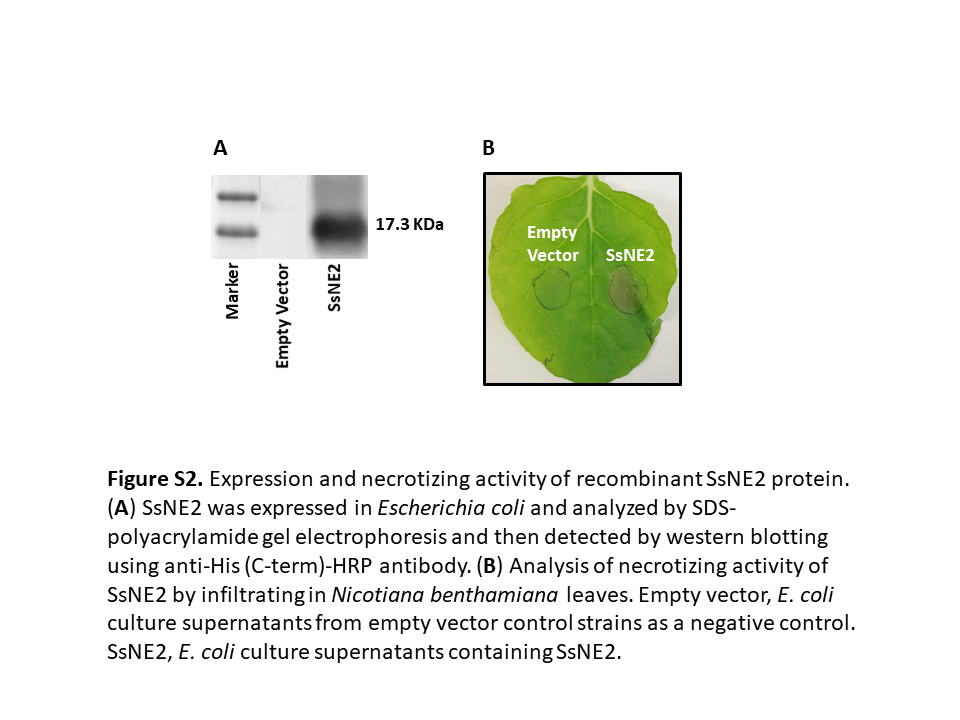

Supplement: Supplementary file 6 [file Image_2.tif]
